# Strigolactones Modulate Cellular Antioxidant Defense Mechanisms to Mitigate Arsenate Toxicity in Rice Shoots

**DOI:** 10.3390/antiox10111815

**Published:** 2021-11-15

**Authors:** Mohammad Golam Mostofa, Chien Van Ha, Md. Mezanur Rahman, Kien Huu Nguyen, Sanjida Sultana Keya, Yasuko Watanabe, Misao Itouga, Abeer Hashem, Elsayed Fathi Abd_Allah, Masayuki Fujita, Lam-Son Phan Tran

**Affiliations:** 1 Department of Biochemistry and Molecular Biology, Bangabandhu Sheikh Mujibur Rahman Agricultural University, Gazipur 1706, Bangladesh; mostofa@bsmrau.edu.bd or; 2 Institute of Genomics for Crop Abiotic Stress Tolerance, Department of Plant and Soil Science, Texas Tech University, Lubbock, TX 79409, USA; chien.ha@ttu.edu (C.V.H.); mdmerahm@ttu.edu (M.M.R.); skeya@ttu.edu (S.S.K.); 3 National Key Laboratory for Plant Cell Biotechnology, Agricultural Genetics Institute, Vietnam Academy of Agricultural Science, Hanoi 100000, Vietnam; kienbio280888@gmail.com; 4 Bioproductivity Informatics Research Team, RIKEN Center for Sustainable Resource Science, Yokohama 230-0045, Japan; yasuko.watanabe@riken.jp; 5 Synthetic Genomics Research Group, RIKEN Center for Sustainable Resource Science, Yokohama 230-0045, Japan; misao.itouga@riken.jp; 6 Botany and Microbiology Department, College of Science, King Saud University, P.O. Box 2460, Riyadh 11451, Saudi Arabia; habeer@ksu.edu.sa; 7 Plant Production Department, College of Food and Agricultural Sciences, King Saud University, P.O. Box 2460, Riyadh 11451, Saudi Arabia; eabdallah@ksu.edu.sa; 8 Laboratory of Plant Stress Responses, Department of Applied Biological Science, Faculty of Agriculture, Kagawa University, Takamatsu 761-0795, Japan; fujita.masayuki@kagawa-u.ac.jp; 9 Institute of Research and Development, Duy Tan University, Da Nang 550000, Vietnam

**Keywords:** arsenate stress, *dwarf* mutants, enzyme activation, glutathione, oxidative damage, rice, strigolactone, vacuolar sequestration

## Abstract

Metalloid contamination, such as arsenic poisoning, poses a significant environmental problem, reducing plant productivity and putting human health at risk. Phytohormones are known to regulate arsenic stress; however, the function of strigolactones (SLs) in arsenic stress tolerance in rice is rarely investigated. Here, we investigated shoot responses of wild-type (WT) and SL-deficient *d10* and *d17* rice mutants under arsenate stress to elucidate SLs’ roles in rice adaptation to arsenic. Under arsenate stress, the *d10* and *d17* mutants displayed severe growth abnormalities, including phenotypic aberrations, chlorosis and biomass loss, relative to WT. Arsenate stress activated the SL-biosynthetic pathway by enhancing the expression of SL-biosynthetic genes *D10* and *D17* in WT shoots. No differences in arsenic levels between WT and SL-biosynthetic mutants were found from Inductively Coupled Plasma-Mass Spectrometry analysis, demonstrating that the greater growth defects of mutant plants did not result from accumulated arsenic in shoots. The *d10* and *d17* plants had higher levels of reactive oxygen species, water loss, electrolyte leakage and membrane damage but lower activities of superoxide dismutase, ascorbate peroxidase, glutathione peroxidase and glutathione *S*-transferase than did the WT, implying that arsenate caused substantial oxidative stress in the SL mutants. Furthermore, WT plants had higher glutathione (GSH) contents and transcript levels of *OsGSH1*, *OsGSH2*, *OsPCS1* and *OsABCC1* in their shoots, indicating an upregulation of GSH-assisted arsenic sequestration into vacuoles. We conclude that arsenate stress activated SL biosynthesis, which led to enhanced arsenate tolerance through the stimulation of cellular antioxidant defense systems and vacuolar sequestration of arsenic, suggesting a novel role for SLs in rice adaptation to arsenic stress. Our findings have significant implications in the development of arsenic-resistant rice varieties for safe and sustainable rice production in arsenic-polluted soils.

## 1. Introduction

**Figure 1:**
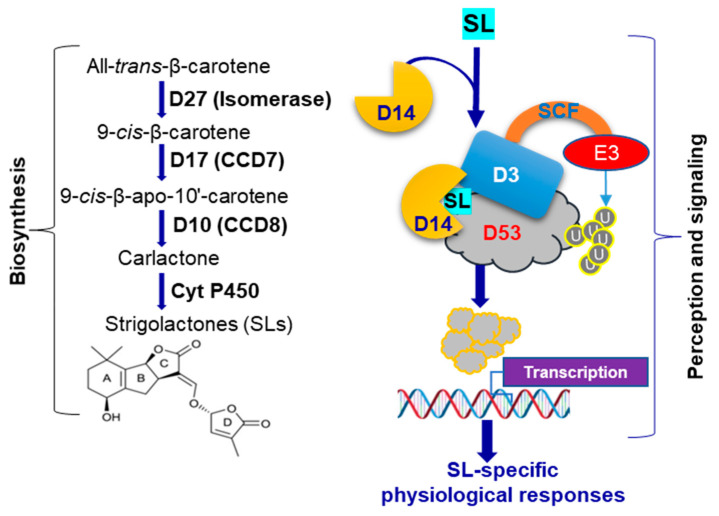
Biosynthesis and signaling of strigolactones (SLs) in rice. SLs are synthesized from all-*trans*-β-carotene by consecutive actions of β-carotene isomerase (*D27*), carotenoid cleavage dioxygenase (CCD)7 (*D17*), CCD8 (*D10*) and cytochrome P450 (Cyt P450) proteins. SLs bind to receptor D14 followed by interaction with D3, leading to the formation of a Skp1-Cullin-F-box (SCF)^D3^ type of E3 ubiquitin ligase complex. D53 is ubiquitinated by this SCF-protein complex, which triggers proteasomal degradation of D53, resulting in SL signaling events for activation of various physiological functions in rice. E, ubiquitin ligase; U, ubiquitin.

Plant growth and developmental processes are regulated by several endogenous molecules, including plant hormones such as strigolactones (SLs) [1,2]. SLs are a new class of phytohormones synthesized from plant pigment carotenoids by the actions of a series of enzymes depending on the types of plant species [1,3,4]. In rice (*Oryza sativa*), three SL-biosynthetic enzymes β-carotene isomerase, carotenoid cleavage dioxygenase (CCD)7 (CCD7) and CCD8 are encoded by *DWARF* (*D*)*27* (*D27*), *D17* and *D10*, respectively [5] (Figure 1). The sequential actions of these three enzymes convert β-carotene into carlactone [5]. Carlactone then undergoes further modification by two cytochrome P450 proteins, namely Os01g0700900 (carlactone oxidase) and Os01g0701400 (orobanchol synthase), to be converted to 4-deoxyprobanchol and orobanchol, respectively [6]. When SLs are absent, transcriptional repressor D53, in cooperation with TOPLESS (TPL)/TOPLESS-RELATED (TPR) repressors, represses downstream signaling of SLs [7]. In the presence of SLs, an α/β hydrolase receptor, namely D14, detects SLs, binds and is activated (Figure 1). SL-activated D14 interacts with the F-box protein D3, leading to the formation of a Skp1-Cullin-F-box (SCF)^D3^ type of E3 ubiquitin ligase complex that acts to degrade D53, thereby enabling the expression of SL-responsive genes for various developmental, physiological and stress survival functions [3,8] (Figure 1). 

SLs were primarily identified as signaling molecules for parasitic seed germination and the establishment of a symbiotic connection between plants and arbuscular mycorrhizal fungi (AMF) in natural environments [9]. Apart from these, SLs can control various developmental traits in both aboveground and belowground parts of plants. SLs positively regulate plant height, leaf senescence, stem thickness, root hair elongation and primary root length, whereas they negatively affect shoot gravitropism and branching, adventitious rooting and lateral root development under normal growth conditions [1,7]. Various loss-of-function studies have used SL-related mutants and synthetic GR24 to demonstrate that SLs played crucial regulatory roles in plant responses to environmental perturbations [10,11,12,13]. For example, SL-deficient *Arabidopsis* (*Arabidopsis thaliana*) and rice mutants, and SL-depleted transgenic *Lotus japonicus* and tomato (*Solanum lycopersicum*) lines showed higher susceptibility to drought than their respective wild-type (WT) plants [13,14,15,16]. Foliar application of GR24 alleviated the deleterious effects of several abiotic stresses, including low light stress in tomato, drought in wheat (*Triticum aestivum*) and salinity in rice [12,17,18]. SL-mediated positive effects on plant tolerance to abiotic stresses mainly pertain to the regulation of various physiological and biochemical processes, including photosynthetic efficiency, leaf senescence, cell wall biogenesis, stomatal closure, flavonoid production, antioxidant defense and nutrient homeostasis [1]. Recently, Qiu et al. [11] reported that GR24 application improved cadmium (Cd) toxicity tolerance in contrasting barley (*Hordeum vulgare*) genotypes by inhibiting Cd uptake, balancing nutrient levels, and activating reactive oxygen species (ROS)-scavenging systems. Although this study pinpoints the likely involvement of SLs in heavy metal tolerance, the genetic and molecular insights into crop tolerance to excessive metal stresses are still obscure. Furthermore, the role of GR24 in stress tolerance should be cautiously interpreted as the sole role of SLs because GR24 is well-known to have both SL and karrikin effects [19]. Thus, to obtain a firm understanding of how SLs potentiate metal-defense networks in plants, it is crucial to use loss-of-function mutants to figure out the SL-modulated genetic basis that governs physiological and biochemical changes for making plants more resilient to metal-induced harsh environments.

Arsenic is the most hazardous metalloid for plant growth and development when it is uptaken in large quantities from the arsenic-contaminated environments [20,21]. Arsenite (As^III^) and arsenate (As^V^) are two inorganic forms of arsenic that are predominantly found in anaerobic and aerobic soils, respectively [22]. In plants, aquaglyceroporins and phosphate transporters, respectively, facilitate the uptake of As^V^ and As^III^ from arsenic-contaminated soils [21,22]. Once accumulated inside the plant tissues, arsenic can inhibit seed germination and seedling establishment, induce oxidative stress, inhibit photosynthesis, suppress growth, and reduce seed quality [23,24,25,26]. To protect themselves, plants deploy several coordinated defense processes, such as restriction of arsenic uptake and transportation, synthesis of arsenic chelating metabolites such as glutathione (GSH) and phytochelatins (PCs), stimulation of antioxidant defense, and vacuolar sequestration of arsenic to reduce arsenic-induced toxicity effects [25,27,28]. 

Agricultural land contamination with arsenic has become a global problem as it has a detrimental influence on all forms of life. Rice is a significant grain crop that feeds half of the world’s population and can be cultivated in a variety of soils, including arsenic-laden soil [23]. As a result, rice and rice-related products account for the majority of arsenic consumed by humans [29]. Moreover, arsenic contamination of soils can escalate the adverse effects of other environmental stresses on rice productivity [30]. Thus, understanding the mechanisms driving arsenic accumulation and detoxification in rice plants is critical for developing future crops that are safe for humans. We have recently reported the involvement of SLs in the regulation of rice root tolerance to excessive As^V^ stress [27]. However, how SLs deal with the arsenic toxicity in aboveground shoots of rice is currently unknown. Because various plant organs evoke diverse responses to metal stress, including arsenic stress [20,31], we were further interested in deciphering the roles of SLs in counteracting the negative effects of arsenic in the shoots of rice plants subjected to As^V^. We evaluated comparative responses of WT and SL-biosynthetic mutants *d10* and *d17* in the presence of various concentrations of As^V^. We have investigated arsenic-metal homeostasis, oxidative stress induction, antioxidant defense response, mineral balance and vacuolar sequestration of arsenic to understand the mechanistic aspects of SL-mediated As^V^ tolerance in rice shoots. Our integrated findings have provided evidence on how SL deficiency exposes rice shoots to vulnerability of arsenic toxicity, and the plausible roles of SLs in alleviation of As^V^ stress in an aboveground organ of rice.

## 2. Materials and Methods

### 2.1. Plant Materials, Plant Cultivation and Stress Treatments

To carry out the current investigation, we used the SL-deficient *dwarf* mutants, namely *d10-1* and *d17-1* (*d10* and *d17* hereafter) and the relevant WT of ‘Shiokari’ background as plant materials [32]. Rice seeds were sterilized with sodium hypochlorite, then allowed for germination in an incubator under dark conditions at 28 ± 2 °C [33,34]. The uniformly germinated rice seeds were raised in a growth chamber under controlled conditions (16-h/8-h light/dark at 25 ± 2 °C; photon flux density of 100 μmol m^−2^ s^−^^1^) in a hydroponic culture (plastic beaker: 250 mL; seedling number in each beaker: 60). The nutrients were supplied to the beakers according to the company’s instructions (Hyponex-all-purpose plant foods, Osaka, Japan), and they were replaced every three days. Fourteen-day-old WT and SL mutant plants were subjected to three levels of sodium arsenate (Na_2_AsO_4_, As^V^) (0, 125 and 250 μM, hereafter referred to as As0, As1 and As2) in the nutrient solution to evaluate the effect of As^V^ stress. Following their exposure to As^V^ treatments, rice plants were allowed to grow for an extra five days in the above-mentioned conditions. Rice plants were harvested after three days of As^V^ treatment (17-day-old plants), and shoot sections were immediately detached for physiological and biochemical parameter measurements, as well as molecular analysis. Each treatment was replicated thrice under the identical experimental circumstances. 

### 2.2. Assessment of Shoot Phenotypes, Shoot Height, Shoot Dry Weight, Photosynthetic Pigment Contents, Electrolyte Leakage and Water Status

The impact of As^V^-induced toxicity on rice performance was first assessed by recording shoot phenotype and measuring shoot height and shoot dry weight (DW) after five days of As^V^ treatments (19-day-old plants). To illustrate the phenotypic changes, a digital camera was used to photograph the entire set of shoots from each treatment. Rice plants’ shoot heights were measured manually on a meter scale and presented in millimeter (mm) seedling^−1^. Shoot DW, electrolyte leakage and water status in terms of relative water content (RWC) of rice plants were determined as per the reported procedures [31]. Extraction of photosynthetic pigments and the collection of supernatants were carried out according to a published protocol [31]. The contents of total chlorophylls (Chls) and carotenoids in rice shoots were calculated based on the formulae reported in [35,36], respectively.

### 2.3. Quantification of the Levels of Arsenic, Phosphorous and Other Minerals in Rice Shoots

After harvesting, shoot samples were immediately freeze-dried for three days, followed by estimation of DW using an analytical balance. Pre-digestion of dried shoot samples were carried out by treatment with 5 mL of strong nitric acid for 1 h at room temperature. Wet-ashing was utilized to fully decompose the organic compounds in samples using a microwave digestion apparatus (Multi Wave 3000; Perkin Elmer, Waltham, MA, USA). The contents of arsenic, phosphorous (P), zinc (Zn), magnesium (Mg) and calcium (Ca) were determined using Inductively Coupled Plasma-Mass Spectrometry (ICP-MS, NexION300, Perkin Elmer, Waltham, MA, USA) following the procedure reported by Itouga et al. [37].

### 2.4. Histochemical Staining of ROS and Cuticle Damage in Rice Leaves

Histochemical detections of superoxide (O_2_**^•^**^−^) and hydrogen peroxide (H_2_O_2_) in rice leaves were carried out using nitroblue tetrazolium (NBT) and 3,3′-diaminobenzidine (DAB), respectively [31]. Toluidine blue (TB) staining was conducted to detect the As^V^-induced cuticle damage in rice leaves following the described protocol [38].

### 2.5. Estimation of H_2_O_2_ and Malondialdehyde Contents in Rice Shoots

The ‘OxiSelectTM Hydrogen Peroxide/Peroxidase Assay Kit (Fluorometric)’ (Cell Biolabs, Inc., San Diego, CA, USA) was used to extract H_2_O_2_ and determine its concentration in rice shoots as described by Nguyen et al. [39]. The technique of Heath and Packer [40] was used to determine the level of malondialdehyde (MDA) in rice shoots. MDA levels in rice shoots were calculated using an extinction coefficient of 155 mM^−1^ cm^−1^.

### 2.6. Extraction and Estimation of Total GSH, Antioxidant Enzyme Activities and Total Soluble Protein Contents

For the extraction of GSH, rice shoots were homogenized in an extraction buffer containing ethylenediaminetetraacetic acid and metaphosphoric acid. The method described by Griffith [41] was adopted to determine total GSH content using a standard graph developed with a series of GSH concentrations. The technique for extracting soluble proteins and preparing supernatants for antioxidant enzyme assays was followed exactly as described previously [31]. Following the procedure of Bradford [42], the Coomassie Protein Assay Kit (ThermoFisher Scientific, Rockford, IL, USA) was utilized to determine soluble protein concentrations in the supernatants. The activity of superoxide dismutase (SOD, EC 1.15.1.1) was measured using a modified version [31] of a previously published technique [43]. The activity of ascorbate peroxidase (APX, EC 1.11.1.11) was measured using the method described in Nakano and Asada [44], while that of glutathione (GSH) reductase (GR, EC 1.6.4.2) according to a prior procedure of Foyer and Halliwell [45]. For the measurement of GSH peroxidase (GPX, EC: 1.11.1.9) and GSH *S*-transferase (GST, EC 2.5.1.18) activities, the comprehensive protocols published in [46,47], respectively, were used.

### 2.7. Gene Expression Analysis

The RNeasy Mini Kit was utilized to extract total RNA from rice leaves, which was then used for cDNA preparation (ReverTra Ace qPCR RT Master Mix, Toyobo, Osaka, Japan). The qRT-PCR assay (Mx3000P qPCR system, Agilent Technologies, Santa Clara, CA, USA) was carried out following the previously published procedure [48]. The transcript levels of various genes in rice shoots were determined using the specific primer pairs shown in Supplementary Table S1. *OsUbiquitin* (*OsUBQ*) was explored as a reference gene in the analysis of the qRT-PCR data.

### 2.8. Data Analysis

The Statistix 10 software was used to carry out a two-way analysis of variance (ANOVA) on all of the data. Arithmetical data are provided as means with standard errors (SEs). For physiological and biochemical parameters, and expression of associated genes, the least significant difference (LSD) post hoc test was carried out to identify significant variations among the treatments (*p* < 0.05). For analyzing expression data of *D10* and *D17* genes, the Student’s *t*-test (** *p* < 0.01) was conducted to identify significant variations among As0, As1 and As2 treatments. 

## 3. Results

### 3.1. SL Deficiency Leads to Severe Arsenic Stress on Rice Growth and Biomass Production

**Figure 2:**
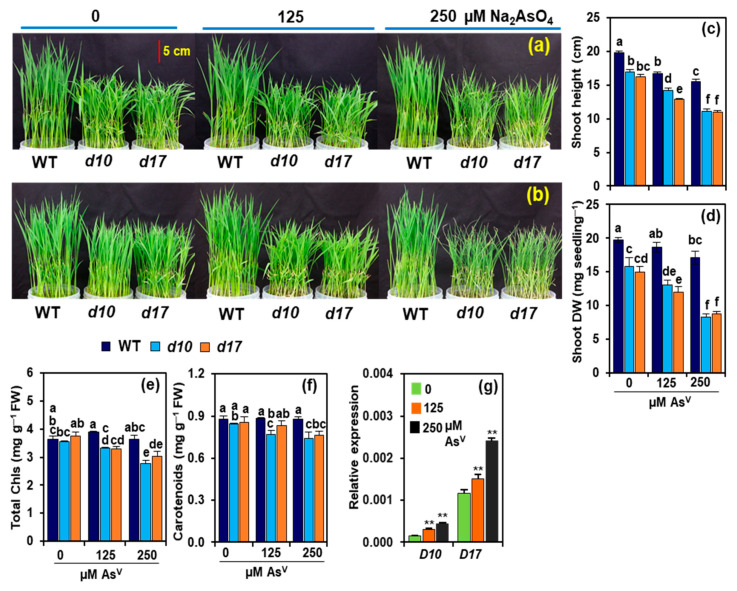
(**a**–**f**) Growth-related features of wild-type (WT) and *d10* and *d17* mutant plants exposed to different concentrations of sodium arsenate (Na_2_AsO_4_; 0, 125 and 250 μM As^V^). Shoot phenotypes were recorded on day 3 (**a**) and day 5 (**b**) of As^V^ treatments. Shoot height (**c**), shoot dry weight (**d**), and the levels of total chlorophylls (**e**) and carotenoids (**f**) were assessed on day 5 of As^V^ treatments. (**g**) Relative expression of *D10* and *D17* genes was examined in the shoots of WT on day 3 of As^V^ treatments. Represented numerical data are the means with standard errors (*n* = 4 biological repeats). Significant differences (*p* < 0.05) among the treatments are denoted by distinct alphabetical letters according to a least significant difference test. Student’s *t*-test (** *p* < 0.01) was conducted to identify significant variations among 0, 125 and 250 μM As^V^ treatments (**g**). Chls, chlorophylls; DW, dry weight; FW, fresh weight.

The shoots of As^V^-exposed *d10* and *d17* mutants showed severe phenotypic aberrations, including rolling and yellowing of leaves, as well as burning of leaf tips, as compared with WT (Figure 2a,b). At As1 and As2 doses, the shoot heights of WT plants were lowered by 15.3 and 21.9%, respectively, when compared with As0 (Figure 2c). By contrast, the shoot heights of *d10* and *d17* plants markedly decreased by 16.5 and 20.4% at As1, and more highly declined by 34.4 and 32.2%, respectively, at As2 in relation to the values at As0 (Figure 2c). Comparable shoot DWs were observed in WT plants under As0 and As1, while it was reduced by 13.4% at As2 (Figure 2d). In contrast, As1 and As2 reduced shoot DW by 17.2 and 47.7% in *d10*, and by 19.9 and 41.3% in *d17* plants, respectively, in comparison with their respective value at As0 (Figure 2d). The photosynthetic pigment data showed that As^V^ stress did not significantly alter the levels of total Chls and carotenoids in WT plants when compared with their respective value at As0 (Figure 2e,f). At As1 and As2, the contents of total Chls were reduced by 6.6 and 21.8%, while the levels of carotenoids were reduced by 8.7 and 12.0% in *d10* mutant, respectively, compared with their respective value at As0 (Figure 2e,f). In *d17* mutant shoots, the levels of total Chls decreased by 11.9 and 19.1% at As1 and As2, respectively, relative to control conditions (Figure 2e). On the other hand, in the shoots of *d17* mutant, carotenoid content remained unaltered at As1, but it showed a significant decline by 11%, at As2 compared with the content observed at As0 (Figure 2f). 

### 3.2. Arsenic Induces the Expression of D10 and D17 in the Shoots of WT Plants

To see whether As^V^ stress affected the expression of genes involved in the SL-biosynthetic pathway, we assessed the transcript levels of *D10* and *D17* in the shoots of WT rice plants after their exposure to As^V^ doses for three days. In contrast to those observed at As0, dramatically elevated transcript levels of *D10* (by 96.6 and 184.1%) and *D17* (by 30.8 and 107.9%, respectively) were noted at As1 and As2 in the shoots of WT plants (Figure 2g). 

### 3.3. SL Deficiency Does Not Alter Arsenic, P and Zn Levels in Rice Shoots

**Figure 3:**
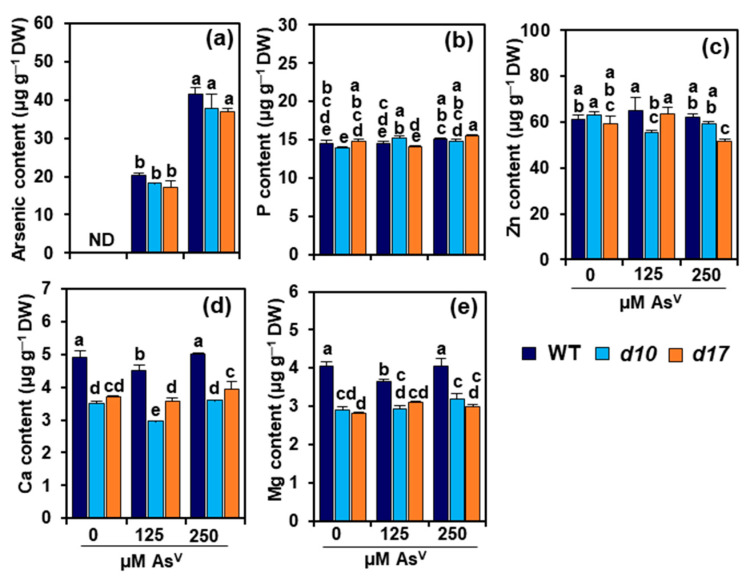
Arsenic and mineral nutrient contents in wild-type (WT) and *d10* and *d17* mutant plants exposed to different concentrations of sodium arsenate (Na_2_AsO_4_; 0, 125 and 250 μM As^V^). The contents of arsenic (**a**), phosphorus (P) (**b**), zinc (Zn) (**c**), calcium (Ca) (**d**) and magnesium (Mg) (**e**) in rice shoots were determined on day 3 of As^V^ treatments. Represented numerical data are the means with standard errors (*n* = 4 biological repeats). Significant differences (*p* < 0.05) among the treatments are denoted by distinct alphabetical letters according to a least significant difference test. DW, dry weight; ND, not detected.

All control plants that were not exposed to As^V^ treatments had no detectable level of arsenic in their shoots (Figure 3a). Arsenic contents steadily and markedly increased in the shoots of WT and SL mutant plants after their exposure to As1 and As2 (Figure 3a). On the other hand, P contents in WT and *d17* shoots remained comparable at As1 and As2 in relation to their levels at As0 (Figure 3b). In *d10* plants, the shoot-P contents displayed an elevation by 10 and 6.3% at As1 and As2, respectively, in comparison with the respective value at As0 (Figure 3b). Nevertheless, there were no significant differences in the arsenic and P contents between the WT and SL-deficient mutants (Figure 3a,b). Zn contents were diminished by 12.3 and 5.8% in *d10* shoots at As1 and As2, respectively, compared with the content recorded at As0 (Figure 3c). In WT and *d17* shoots, Zn contents remained nearly unaltered at As0, As1 and As2 treatments (Figure 3c). In general, SL deficiency did not significantly alter Zn contents under both As^V^ free and As^V^ stress conditions (Figure 3c).

### 3.4. SL Deficiency Alters Ca and Mg Contents in Rice Shoots

In comparison with As0, a significant reduction of Ca content was recorded in the shoots of WT (by 8.1%) and *d10* (by 15.3%) at As1; however, comparable levels of Ca were observed in the shoots of the two genotypes at As2 (Figure 3d). No significant changes in Ca level were observed in the shoots of As^V^-stressed *d17* plants at both As1 and As2 compared with that at As0 (Figure 3d). In relation to As0, As1 displayed a notable decrease (by 10.0%), while As2 showed no significant change in the level of Mg in WT shoots (Figure 3e). Nonetheless, Mg levels remained unchanged in the shoots of mutant plants in responses to As1 and As2 when contrasted with their respective levels obtained at As0 (Figure 3e). Interestingly, under control conditions (As0), WT shoots showed significantly higher contents of Ca and Mg than *d10* and *d17* shoots, and this elevated trend of shoot Ca and Mg contents in WT sustained even under the As^V^ treatments (As1 and As2) (Figure 3d,e). 

### 3.5. SL Deficiency Evokes Arsenic-Induced Oxidative Stress, Cuticle Damage, Electrolyte Leakage and Water Loss

**Figure 4:**
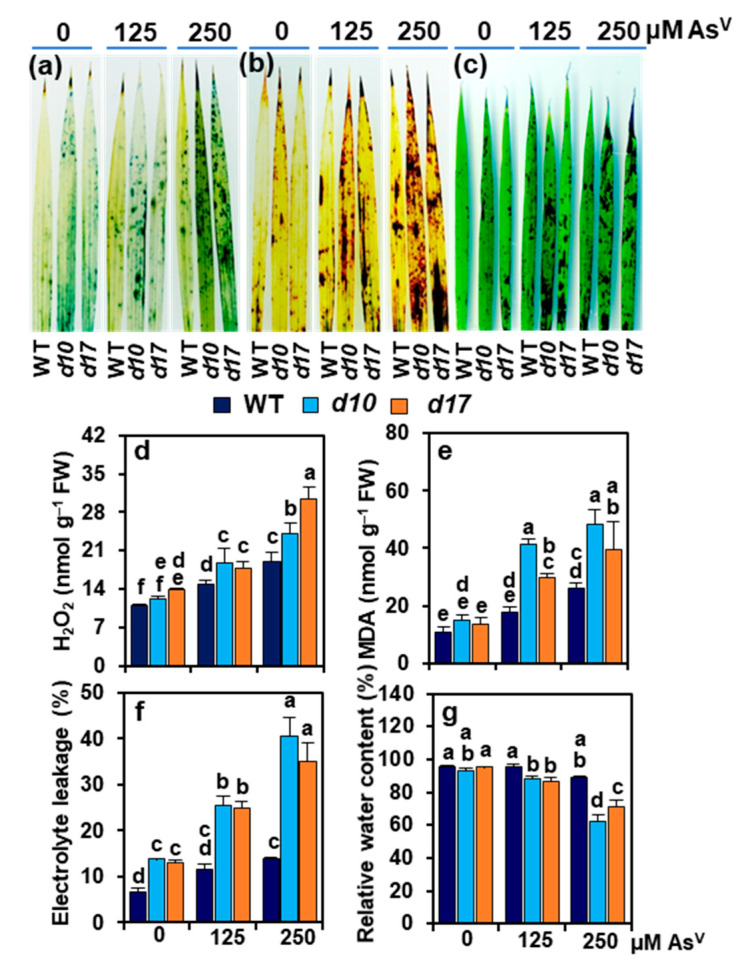
(**a**–**g**) Oxidative stress-related parameters in wild-type (WT) and *d10* and *d17* mutant plants exposed to different concentrations of sodium arsenate (Na_2_AsO_4_; 0, 125 and 250 μM As^V^). (**a**–**c**) Leaf staining for detection of superoxide (O_2_**^•^**^−^) by nitroblue tetrazolium (**a**), hydrogen peroxide (H_2_O_2_) by diaminobenzidine (**b**), and cuticle damage by toluidine blue (**c**). (**d**–**g**) The levels of H_2_O_2_ (**d**), malondialdehyde (MDA), electrolyte leakage (**f**) and relative water contents (**g**) in the shoots of the three genotypes were recorded on day 3 of As^V^ treatments. Represented numerical data are the means with standard errors (*n* = 3 biological repeats). Significant differences (*p* < 0.05) among the treatments are denoted by distinct alphabetical letters according to a least significant difference test. FW, fresh weight.

NBT and DAB staining analyses showed that the leaves of *d10* and *d17* mutants developed more deep blue spots and dark brown spots than WT plants under As^V^ stress, indicating that such stress induced higher accumulation of O_2_**^•^**^−^ and H_2_O_2_, respectively, in the SL-deficient mutants than WT (Figure 4a,b). Similarly, TB staining showed that the leaves of SL-depleted mutant plants exhibited more intense dark spots than WT under As^V^ stress, which was an indication of greater cuticle damage by SL deficiency (Figure 4c). 

In the shoots of WT, a significant increase in H_2_O_2_ was noted by 35.9 and 72.7% at As1 and As2, respectively, over the value found at As0 (Figure 4d). This rise range was even higher in the SL-deficient mutants; specifically, by 56.3 and 99.0% in *d10*, and 29.0 and 121.1% in *d17* shoots at As1 and As2, respectively, when compared with the corresponding values at As0 (Figure 4d). In comparison with As0, MDA contents increased by 62.8 and 141.8% at As1 and As2, respectively, in the shoots of WT. On the other hand, more highly increased MDA contents were noted in the SL-deficient mutants; namely, by 174.2 and 221.7% in *d10* shoots and 116.2 and 188.5% in *d17* shoots at As1 and As2, respectively, over the control values at As0 (Figure 4e). At As1 and As2, WT shoots showed significant increases by 75.3 and 107.2%, respectively, in EL relative to that at As0 (Figure 4f). EL was more severe by SL deficiency, showing the increased values of 82.3 and 90.4% at As1, and 189.7 and 169.1% at As2 in the shoots of *d10* and *d17* mutant plants, respectively, in contrast to their data at As0 (Figure 4f). WT plants had comparable RWC in the shoots at all levels of As^V^ treatments, while SL-deficient mutants displayed lower RWC than WT at As1 and As2 (Figure 4g). In *d10* plants, RWC remained unaffected at As1; however, it showed a reduction by 33.3% at As2 in relation to As0 (Figure 4g). In *d17* plants, the RWC was reduced by 8.8 and 25.2% at As1 and As2, respectively, in comparison with As0 (Figure 4g).

### 3.6. SL Deficiency Compromises Antioxidant Defense System under As^V^ Stress 

**Figure 5:**
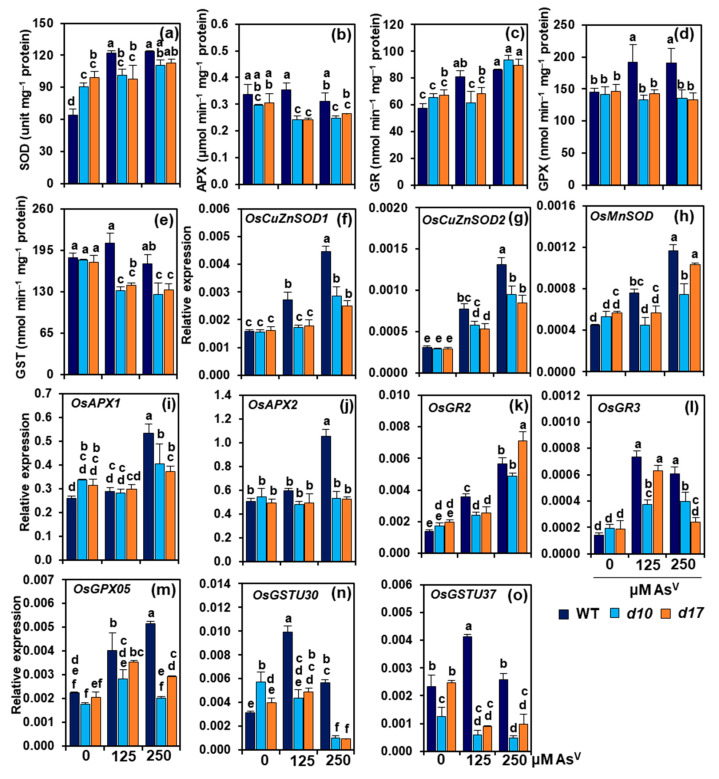
(**a**–**o**) Antioxidant enzyme activities and transcript levels of related genes in the shoots of wild-type (WT) and *d10* and *d17* mutant plants exposed to different concentrations of sodium arsenate (Na_2_AsO_4_; 0, 125 and 250 μM As^V^). (**a**–**e**) Superoxide dismutase (SOD) (**a**), ascorbate peroxidase (APX) (**b**), glutathione reductase (GR) (**c**), glutathione peroxidase (GPX) (**d**) and glutathione *S*-transferase (GST) (**e**) activities in the shoots of three genotypes on day 3 of As^V^ treatments. (**f**–**o**) Relative expression of biosynthetic genes of SOD (*OsCuZnSOD1*, *OsCuZnSOD2* and *OsMnSOD*) (**f**–**h**), APX (*OsAPX1* and *OsAPX2*) (**i**,**j**), GR (*OsGR2* and *OsGR3*) (**k**,**l**), GPX (*OsGPX05*) (**m**) and GST (*OsGSTU30* and *OsGSTU37*) (**n**,**o**) enzymes in the shoots of WT, *d10* and *d17* on day 3 of the As^V^ treatments. Represented numerical data are the means with standard errors (*n* = 3 biological repeats). Significant differences (*p* < 0.05) among the treatments are denoted by distinct alphabetical letters according to a least significant difference test.

In comparison with As0, As1 and As2 enhanced SOD activities by 91 and 92.7%, respectively, in the shoots of WT (Figure 5a). On the other hand, SOD activities in *d10* shoots did not show significant alteration at As1 but an increase by 22.4% at As2, when compared with As0. In *d17* shoots, SOD activities remained comparable at both levels of As^V^ when contrasted with As0 (Figure 5a). APX activities showed insignificant change at As1 but a decrease by 7.6% in WT shoots at As2 relative to As0. However, in comparison with As0, As1 and As2 decreased APX activities by 18.2 and 16.1% in *d10* shoots, and 20.7 and 13.6% in *d17* shoots, respectively (Figure 5b). WT plants treated with As1 and As2 showed an increase in GR activity by 40.2 and 49.2%, respectively, over the control value obtained at As0 (Figure 5c). By contrast, shoot-GR activities in *d10* and *d17* plants were not altered at As1 but enhanced by 42.6 and 32.7% at As2, respectively, when contrasted with the data at As0 (Figure 5c). WT plants treated with As1 and As2 showed significant increases in shoot-GPX activity, by 32.3 and 31.3%, respectively, over the data at As0 (Figure 5d). However, GPX activities showed an unaltered trend in the shoots of *d10* and *d17* plants raised at any levels of As^V^ (Figure 5d). In WT shoots, GST activities remained unaltered at both As1 and As2 doses when compared with As0 (Figure 5e). On the other hand, As1 and As2 decreased GST activities in shoots of *d10* plants by 26.4 and 30.0%, and *d17* plants by 20.4 and 24.9%, respectively, in comparison with As0 (Figure 5e). 

In WT shoots, the transcript levels of SOD-encoding gene *OsCuZnSOD1* were notably elevated by 69.9 and 181.2% at As1 and As2, respectively, whereas in *d10* and *d17* shoots, its expression levels increased by 82.8% and 55.7%, respectively, only at As2, in relation to As0 (Figure 5f). The *OsCuZnSOD2* transcripts were more remarkably enhanced in WT shoots (by 151.6 and 329.2%, respectively) than in *d10* (by 96.7 and 223.0%) and *d17* (by 80 and 189.9%) shoots at both As1 and As2, when compared with As0 (Figure 5g). Likewise, the expression levels of *OsMnSOD* more highly increased in the shoots of WT (by 69.4 and 160.8% at As1 and As2, respectively) than in *d10* (by 39.9% at As2) and *d17* plants (by 81.9% at As2) (Figure 5h). In comparison with As0, the expression levels of APX-encoding genes *APX1* and *APX2* remained unaltered at As1 in WT, *d10* and *d17* shoots. However, at As2, the transcript levels of *APX1* and *APX2* showed a greater enhancement, by 106.9 and 109.9%, respectively, in WT compared with the respective values obtained at As0. Nonetheless, the *d10* and *d17* shoots did not show significant alteration in *APX1* and *APX2* transcripts at both As1 and As2 compared with As0 (Figure 5i,j). 

The transcript levels of *OsGR2* in the shoots of WT plants dramatically increased by 156.9 and 306.7% at As1 and As2 over the value found at As0 (Figure 5k). However, *OsGR2* transcript levels in *d10* and *d17* plants did not alter at As1 but significantly increased by 180.3 and 258.0% at As2, respectively, compared with the data at As0 (Figure 5k). *OsGR3* expression levels more highly increased in WT shoots (by 411.3 and 323.8%, respectively) than in *d10* (by 91.2 and 103.8%) and *d17* shoots (by 240.3% and 30.8%) in responses to As1 and As2 compared with As0 (Figure 5l). The expression levels of the GPX-encoding gene *OsGPX05* improved by 78.6 and 128.8% in WT, and 71.8 and 42.7% in *d17* shoots at As1 and As2, respectively, in comparison with As0 (Figure 5m). Nevertheless, in *d10* shoots, the transcript level of *OsGPX05* was raised by 61.3% at As1, but it was not significantly altered at As2 compared with As0 (Figure 5m). 

The expression levels of GST-encoding gene *OsGSTU30* in WT shoots increased by 214.9 and 79.2% in response to As1 and As2, respectively, in relation to As0 (Figure 5n). However, the *OsGSTU30* transcript levels in *d10* shoots dramatically decreased in response to As1 (by 23.9%) and As2 (by 82.2%) when compared with As0 (Figure 5n). Additionally, *OsGSTU30* expression in *d17* shoots displayed an insignificant increase at As1, but its transcript level drastically declined by 76.8% at As2, in relation to As0 (Figure 5n). When compared with As0, *OsGSTU37* expression level increased by 78.1% at As1 but its transcript level remained unchanged at As2 in WT shoots (Figure 5o). In *d10* shoots, *OsGSTU37* expression levels declined upon exposure to As^V^ stress, but this decline was significant only at As2 (by 61.4%) when contrasted with As0 (Figure 5o). Furthermore, the expression levels of shoot-*OsGSTU37* decreased by 63.8 and 60.3% in *d17* plants at As1 and As2, respectively, when compared with As0 (Figure 5o). 

### 3.7. SL Deficiency Negatively Affects GSH-Assisted Arsenic Detoxification 

**Figure 6:**
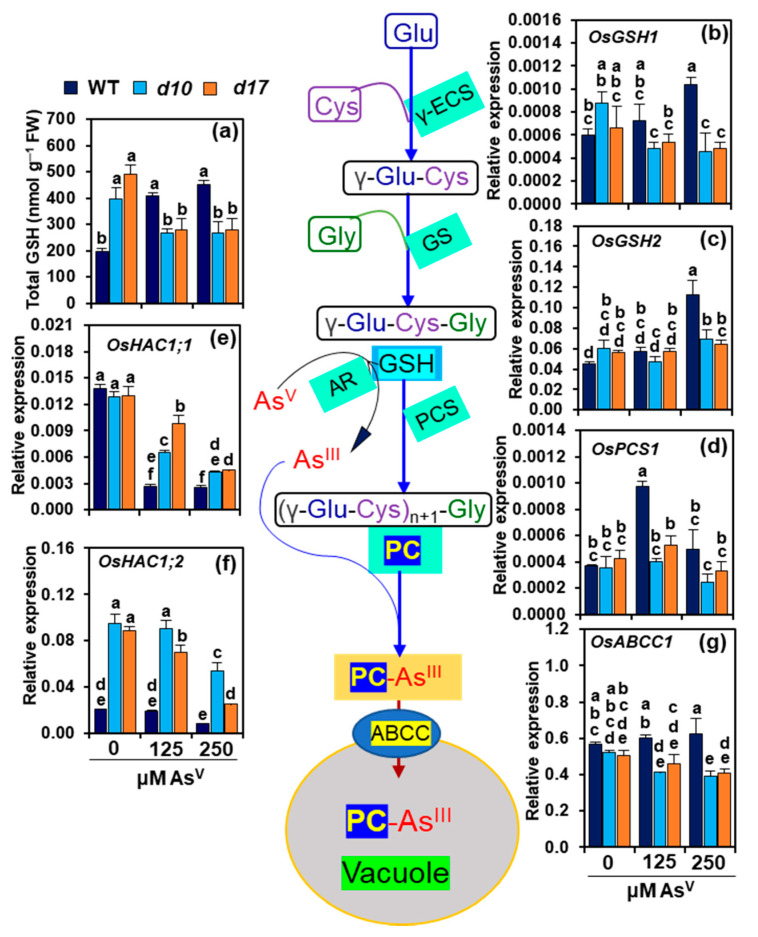
(**a**–**g**) Glutathione (GSH) biosynthesis and GSH-assisted arsenic detoxification in the shoots of wild-type (WT) and *d10* and *d17* mutant plants exposed to different concentrations of sodium arsenate (Na_2_AsO_4_; 0, 125 and 250 μM As^V^). Total GSH content (**a**), and relative expression of genes involved in the biosyntheses of GSH (*OsGSH1* (**b**) and *OsGSH2* (**c**)), phytochelatin synthase, PCS (*OsPCS1* (**d**)), arsenate reductase, AR (*OsHAC1;1* (**e**) and *OsHAC1;2* (**f**)) and C-type ATP-binding cassette, ABCC (*OsABCC1* (**g**)) were determined in the shoots of three genotypes on day 3 of As^V^ treatments. Represented numerical data are the means with standard errors (*n* = 3 biological repeats). Significant differences (*p* < 0.05) among the treatments are denoted by distinct alphabetical letters according to a least significant difference test. As^III^, arsenite; Cys, cysteine; FW, fresh weight; Glu, glutamate; Gly, glycine; γ-ECS, γ-glutamyl cysteine synthetase; GS, glutathione synthetase, HAC, high arsenate content; PC, phytochelatin.

In WT plants, As1 and As2 led to a considerable rise in shoot contents of total GSH by 104.8 and 127.1%, respectively, when compared with As0 (Figure 6a). On the other hand, SL-depleted mutants stressed with As1 and As2 showed a substantial decline in the total GSH levels by 32.4 and 33.1% in *d10* shoots, and 43.2 and 42.6%, respectively, in *d17* shoots compared with the respective data at As0 (Figure 6a). We also investigated the expression of genes involved in the biosynthesis of GSH (*γ-glutamyl cysteine synthetase 1*, *OsGSH1* and *glutathione synthetase 2*, *OsGSH2*), phytochelatin (*phytochelatin synthase 1*, *OsPCS1*), arsenate reductase (*high arsenate content* 1, *OsHAC1;1* and *high arsenate content* 2, *OsHAC1;2*) and As^III^-PC ABC transporter (*C-type ATP-binding cassette* (*ABC*) *transporter*, *OsABCC1*) in the shoots of all studied genotypes (Figure 6b–g). At As1, WT shoots had no significant changes in *OsGSH1* and *OsGSH2* transcript levels; however, at As2, their transcript levels were considerably raised by 74.75 and 151.9%, respectively, as compared with As0 (Figure 6b,c). In *d10* shoots, while *OsGSH1* expression levels were significantly reduced by 45.1 and 48.3% at As1 and As2, respectively, those of *OsGSH2* remained unaltered under both two stress conditions in comparison with As0. The *d17* shoots, on the other hand, showed no significant changes in *OsGSH1* and *OsGSH2* transcript levels at both As1 and As2 compared with As0 (Figure 6b,c).

In the shoots of WT plants, *OsPCS1* expression levels increased by 163.1 and 34.4% at As1 and As2, respectively, relative to the data obtained at As0. However, *d10* and *d17* shoots did not show significant alteration in the expression levels of *OsPCS1* at As1 and As2 when compared with As0 (Figure 6d). The transcript levels of *OsHAC1;1* displayed significant attenuation in the shoots of WT (by 80.4 and 81.7%), *d10* (by 49.8 and 66.8%) and *d17* (by 24.8 and 65.1%, respectively) at As1 and As2 when compared with the respective values at As0 (Figure 6e). The expression levels of *OsHAC1;2* remained unaltered in WT shoots at both As1 and As2 compared with As0. In comparison with As0, As2 decreased the transcript levels of *OsHAC1;2* by 43.2 and 71.8% in *d10* and *d17* mutants, while As1 showed a decrease by 21.2% in *d17* mutant only (Figure 6f). The expression levels of *OsABCC1* remained at the control As0 level in WT shoots at As1 and As2. However, As1 and As2 caused a reduction in *OsABCC1* expression levels in the shoots of *d10* (by 21.2 and 24.8%) and *d17* (by 9.3 and 19.3%, respectively) compared with As0 (Figure 6g).

## 4. Discussion

In a recent work, it was found that SLs helped rice roots cope with As^V^ stress by limiting arsenic uptake and accumulation, while also reducing Pi uptake [27]. Following this report, we looked at the amounts of arsenic and P in rice shoots and discovered that despite having equal levels of arsenic and P, SL-depleted mutants (*d10* and *d17*) displayed more damage in their shoots than WT (Figure 1a,b and Figure 2a,b). We were then curious as to why these mutants were more vulnerable to arsenic and what mechanisms might be responsible for WT’s superior performance under high As^V^ stress circumstances. Because arsenic is a non-essential, hazardous element for plant growth and metabolism, its accumulation in tissues, even at low concentrations, can impair developmental processes in crop plants, including rice, wheat and mustard (*Brassica juncea*) [49,50,51]. Indeed, it was noticed that both the WT and SL-depleted mutants displayed growth defects in an As^V^ concentration-dependent manner. However, phenotypic abnormalities, growth reduction, and biomass loss were far more severe in SL mutants than WT (Figure 2a–d). These findings showed that *d10* and *d17* shoots were more sensitive to As^V^ than WT shoots, indicating that SL depletion led to a more arsenic-susceptible phenotype of rice shoots. It was also found that As^V^ addition resulted in a greater reduction of photosynthetic pigment contents in both *d10* and *d17* plants, which supports the SL-deficient mutant plants’ inferior growth performance when compared with WT (Figure 2e,f). These findings corroborated numerous earlier studies in which photosynthetic components, such as Chls and carotenoids, were the primary targets of arsenic toxicity [26,52,53]. 

**Figure 7:**
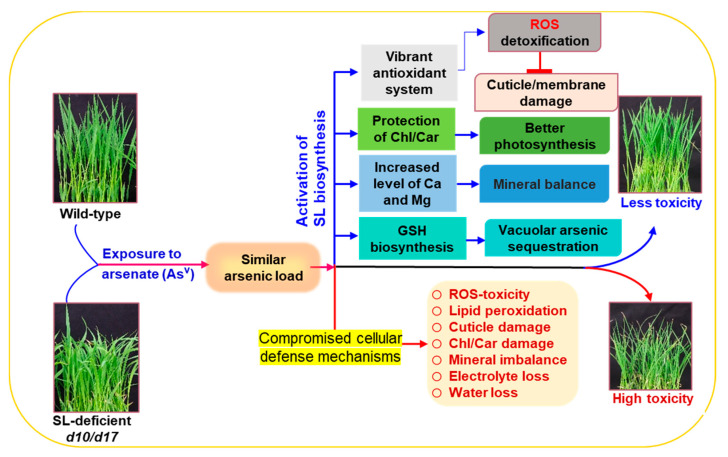
Illustration of strigolactones (SLs)-mediated resistance mechanisms against arsenate toxicity in the shoots of rice plants. Exposure of wild-type (WT) and SL-depleted *d10* and *d17* mutants to arsenate (As^V^) stress resulted in a similar level of arsenic in the shoots of both WT and SL-deficient mutant genotypes. Despite having similar levels of arsenic in the shoots, SL mutants suffered severe As^V^ toxicity because of compromised cellular defense mechanisms. On the other hand, As^V^ stress-activated SL biosynthesis (e.g., As^V^-induced expression of *D10* and *D17*) in WT shoots, leading to stimulation of several cellular defense strategies, which helped WT plants to reduce As^V^ toxicity and perform better under excessive As^V^ stress.

To check whether As^V^ stress had any modulatory effect on SL biosynthesis, the expression patterns of SL-biosynthetic genes *D10* and *D17* were investigated in the shoots of WT rice under all doses of As^V^ (Figure 2g). It was found that exposure of WT plants to As^V^ increased the amounts of *D10* and *D17* transcripts, suggesting that the SL-biosynthetic pathway was favorably activated in WT shoots by As^V^, as it was observed in the As^V^-stressed rice roots earlier [27]. Elevated transcript levels of SL-biosynthetic genes have been reported in rice stems (e.g., *D27*, *D17* and *D10*) under drought stress, and in *Arabidopsis* leaves (e.g., *MORE AXILLARY BRANCHING* (*MAX*)*3* (*MAX3*) and *MAX4*, the orthologous genes of rice *D17* and *D10*, respectively) following dehydration treatment [13,54], indicating that different types of abiotic stresses generally induce the expression of SL-biosynthetic genes in plant shoots (Figure 2g and Figure 7). These results, along with those of others, point to the involvement of SLs in the aboveground organs of many plant species to aid their survival under stressful situations.

Next, we searched for a connection between As^V^-induced damage and arsenic levels in the shoots of WT and SL-deficient mutant plants. Surprisingly, no significant variation in shoot-arsenic content among WT, *d10* and *d17* plants was found, although SL-depleted mutants appeared to be more negatively impacted by As^V^ than WT (Figure 3a). These findings raised the question of why SL-deficient mutants were sensitive to As^V^, while the shoots of both WT and mutants accumulated comparable amounts of arsenic. We then checked whether As^V^ exposure affected the homeostasis of other minerals in the shoots, such as P, Zn, Ca and Mg (Figure 3b–e). Despite increasing the amount of As^V^ doses, P, Zn, Ca and Mg contents remained relatively stable at the control level in WT shoots. On the other hand, *d10* and *d17* shoots contained almost equal levels of P and Zn but similarly lower levels of Ca and Mg when compared with their respective content in WT shoots at both normal and As^V^ stress conditions (Figure 3b–e). These results pinpointed that SL deficiency did not affect the homeostases of P and Zn in the shoots under As^V^ stress conditions. However, the reduced levels of Ca and Mg in *d10* and *d17* shoots under normal conditions suggest that SL deficiency causes an imbalance in their homeostasis. Thus, we inferred that reduced basal Ca and Mg levels may be one of the factors contributing to the poor growth performance of *d10* and *d17* mutants when being challenged with excessive As^V^ (Figure 3d,e and Figure 7). Under both normal and As^V^ stress conditions, P and Zn showed comparable contents in the shoots of both the mutants and WT, whereas Ca and Mg exhibited similarly lower levels in the shoots of the mutants than WT (Figure 3b–e). These data suggest that As^V^ stress does not have any effect on the contents of all four investigated elements.

Excess arsenic in plant tissues has many detrimental effects on cellular metabolism. For example, As^V^ can substitute P from key cellular metabolites such as ATP due to its analogy with P, causing energy metabolism to be disrupted [55]. As^III^ can bind to the sulfhydryl (SH) groups of different sulfur-containing peptides and proteins, preventing them from forming active conformation and functioning as part of the cellular defense system [56]. Both types of arsenic (As^V^ and As^III^) can disrupt the electron transport system in chloroplasts and mitochondria, which are the primary sites of ROS production in cells [57,58]. The transformation of As^V^ to As^III^ can also directly contribute to ROS production via a Haber–Weiss reaction [59]. As a result, ROS generation and oxidative damage in plant tissues are considered typical hallmarks of arsenic-induced toxic effects. In the current study, we concentrated on how WT and SL-deficient mutant plants dealt with ROS and their detoxification systems under arsenic stress. Results revealed that the mutant plants’ shoots accumulated large amounts of O_2_**^•^**^−^ and H_2_O_2_ in response to As^V^ stress (Figure 4a,b,d). This increased production of ROS was positively correlated with the increased levels of lipid peroxidation product MDA and cuticle damage in the shoots of SL mutant plants exposed to As^V^ stress (Figure 4c,e). Furthermore, the increased levels of MDA and cuticle damage by As^V^ stress corresponded with increased electrolyte leakage and water loss in *d10* and *d17* shoots (Figure 4f,g). These findings clearly showed that As^V^ treatments caused considerable oxidative damage in SL-deficient mutant plants, confirming that ROS-induced oxidative stress is one of As^V^’s lethal effects in rice (Figure 7), as also observed in many arsenic-susceptible cultivars such as maize (*Zea mays*) and barley [49,52,60].

The cellular antioxidant system comprising both enzymatic and non-enzymatic arsenals plays crucial roles in relieving the oxidative burden in plant tissues under stressful conditions. To investigate the SLs’ influence on the antioxidant system at the genetic level, the activities of several key antioxidant enzymes, including APX, SOD, GR, GST and GPX, were evaluated in the shoots of both WT and SL-deficient plants under normal and As^V^ stress conditions (Figure 5a–e). During As^V^ stress, the SL-deficient mutants had considerably lower activities of SOD, APX, GR (except at As2), GST and GPX in their shoots than WT plants. qRT-PCR analysis of the expression of relevant genes associated with the biosyntheses of the studied enzymes also displayed that the transcript levels of *OsCuZnSOD1*, *OsCuZnSOD2* and *OsMnSOD* (encoding SOD), *OsAPX1* and *OsAPX2* (encoding APX), *OsGPX05* (encoding GPX), and *OsGSTU30* and *OsGSTU37* (encoding GST) positively correlated with the suppressed activities of respective enzymes in the SL-depleted mutant plants (Figure 5f–m). These findings highlighted that the compromised antioxidant systems in SL-deficient mutant plants were unable to handle the excessive ROS induced by As^V^, thereby these mutants suffered from severe oxidative stress. These results clearly demonstrated the likely roles of SLs in boosting the plant antioxidant defense system against arsenic-induced ROS (Figure 7).

GSH is a powerful non-enzymatic antioxidant that plays various critical roles in plant protection against heavy metal toxicity. GSH, either directly or by the formation of PCs, can aid in the chelation of As^III^ for vacuolar sequestration in the cells [25]. Ideally, PC synthase (PCS) catalyzes the synthesis of PCs from GSH followed by complexation with As^III^ (As^III^-PC complex), which is then sequestrated into the vacuoles by ABCC transporter [20] (Figure 6). GSH can also assist GSH-dependent enzymes such as GPX and GST to detoxify toxic aldehydes generated by ROS-induced lipid oxidation [61]. Thus, any depletion in cellular GSH levels might expose plant cells to vulnerable situations, especially under metal-induced adverse conditions. The present study critically evaluated the GSH-assisted arsenic detoxification by estimating GSH content and analyzing the expression levels of several associated genes, including *OsGSH1*, *OsGSH2*, *OsHAC1;1*, *OsHAC1;2*, *OsPCS1* and *OsABCC1* (Figure 6a–g). It was observed that As^V^ stress resulted in a significant reduction of GSH in the shoots of both SL-deficient mutants. It is plausible that As^V^ stress-mediated inhibition of the expression of GSH biosynthesis-associated genes *OsGSH1* and *OsGSH2* was responsible for the decrease in GSH pool in the shoots of the As^V^-stressed SL-deficient mutants (Figure 6b,c). AR catalyzes the conversion of As^V^ to As^III^ by utilizing GSH [62]. High expression levels of AR-encoding genes *OsHAC1;1* and *OsHAC1;2* also indicated that reduced levels of GSH might have resulted from the consumption of a large quantity of GSH by AR for the transformation of As^V^ to As^III^ in the shoots of the mutants (Figure 6e,f). It is worth noting that As^III^ is far more destructive to cells than As^V^ [63]. We assumed that the decreased transcript levels of *OsPCS1* and *OsABCC1* genes resulted in a reduced complexation of As^III^ with PC and sequestration of As^III^-PC to vacuoles, respectively, leading to increased As^III^ toxicity in the mutant shoots (Figure 2a–f and Figure 6d,g). On the other hand, the elevated GSH content and increased expression levels of *OsPCS1* and *OsABCC1* in WT shoots suggest that GSH-assisted As^III^ detoxification was well executed to reduce arsenic toxicity at the cellular level (Figure 1b–e, Figure 4d,g and Figure 7). 

## 5. Conclusions

Based on the results, we conclude that SL deficiency did not influence the level of arsenic in the shoots of rice plants, and high susceptibility of SL-deficient mutants to As^V^ resulted from the weakened cellular defense mechanisms responsible for arsenic detoxification (Figure 7). The identified most critical defense strategies that contributed to better resistance of WT against As^V^ stress include (i) protection of photosynthetic pigments and high basal levels of minerals (e.g., Ca and Mg) for better growth response, (ii) enhanced detoxification of ROS through a stimulated antioxidant system for safeguarding membrane structure and cuticles, and (iii) increased biosynthesis of GSH for efficient GSH-assisted arsenic detoxification (Figure 7). Overall, these findings revealed that SLs can function as an important growth regulator for enhancing arsenic detoxification mechanisms, including stimulation of cellular antioxidant defense systems and vacuolar sequestration of arsenic in rice shoots, thereby assisting rice plants to overcome excessive arsenic-induced toxic consequences at the cellular level.

## Data Availability

Data is contained within the article and Supplementary Material.

## Supplementary Materials

The following are available online at https://www.mdpi.com/article/10.3390/antiox10111815/s1, Table S1: Primer sequences of reference and target genes used in the current study.
