# Supplementary material for: Strigolactones Modulate Cellular Antioxidant Defense Mechanisms to Mitigate Arsenate Toxicity in Rice Shoots
Source: Antioxidants (Basel). 2021 Nov 15;10(11):1815. doi: 10.3390/antiox10111815 (PMC8614715; doi:10.3390/antiox10111815)
Supplement: Supplementary file 1 [file antioxidants-10-01815-s001.zip › antioxidants-1412120-supplementary.pdf]

Supplementary data:

Supplementary Table S1. Primer sequences of reference and target genes used in the current study

**Purpose**	**Name**	**Forward (5'-3')**	**Reverse (5'-3')**
**Reference gene**	*OsUBQ*	GACGGACGCACCCTGGCTGACTAC	TGCTGCCAATTACCATATACCACGAC
**Target genes**	*D10*	CTGTACAAGTTCGAGTGGCACC	CCTCGTCCGTCTCCTCGTAC
	D17	CCTCGTCCAGAAGCGTGAG	TAGTGGGTGTCGGTGAAGGC
	OsHAC1;1	TGAACAAGGGCCATCTACAC	GACGAGAACTGCTCCACAAA
	OsHAC1;2	TAGCATCTGCCGATCTCATA	GAGGTTTATTCACCGCAAGG
	OsGSH1	TGTCATTTCGGGTCTCTTCC	AAGGGCAAACCTCAGGAAAT
	OsGSH2	GCTGGTCAGACTCCAGAAGG	CCGCAAATAGGCTCCAAATA
	OsPCS1	TCGACGATTTCTGTTTGCTG	GTTCATGGCTTCCCAAAGAA
	OsABCC1	AACAGTGGCTTATGTTCCTCAAG	AACTCCTCTTTCTCCAATCTCTG
	OsCuZnSOD1	ATGCGTCTGTGTTGCTTCTG	CCATCTCCCTCTTGGACAAA
	OsCuZnSOD2	GCGGTGAGTGACTTGGTTTT	TACGAGCGAACATGAACAGC
	OsMnSOD2	TTTTGTGGGTGGGAAATCAT	CACAGCAGCTCCATCCTACA
	OsAPX1	CCAAGGGTTCTGACCACCTA	CAAGGTCCCTCAAAACCAGA
	OsAPX2	TCAAGGACCAACTTCCCATC	AGGGTGTGACCACCAGAAAG
	OsGR2	AAGTGATTCCTCGCGCTAAA	CTCGTCCTTGAGCATCTTCC
	OsGR3	ATATGGGCTGTGGGTGATGT	TGAAGCATCAAAATCGCAAG
	OsGPX05	GATCTGGACCTCCTCACTCG	CCTCATCCACCCTCTTTCAA
	OsGSTU30	GAGGATCTCCGCAACAAGAG	CGATATAGGCGGTCCAGAAG
	OsGSTU37	GCTGAGTCTGCCGGATAGAG	ATTTATTCCCAGGTGGCACA
